# Elevated Levels of Specific Carotenoids During Acclimation to Strong Light Protect the Repair of Photosystem II in *Synechocystis* sp. PCC 6803

**DOI:** 10.3389/fpls.2020.01030

**Published:** 2020-07-07

**Authors:** Taichi Izuhara, Ikumi Kaihatsu, Haruhiko Jimbo, Shinichi Takaichi, Yoshitaka Nishiyama

**Affiliations:** ^1^ Department of Biochemistry and Molecular Biology, Graduate School of Science and Engineering, Saitama University, Saitama, Japan; ^2^ Graduate School of Art and Sciences, The University of Tokyo, Tokyo, Japan; ^3^ Department of Molecular Microbiology, Faculty of Life Sciences, Tokyo University of Agriculture, Tokyo, Japan

**Keywords:** acclimation, carotenoids, singlet oxygen, photoinhibition, photosystem II

## Abstract

The tolerance of photosynthesis to strong light increases in photosynthetic organisms during acclimation to strong light. We investigated the role of carotenoids in the protection of photosystem II (PSII) from photoinhibition after acclimation to strong light in the cyanobacterium *Synechocystis* sp. PCC 6803. In cells that had been grown under strong light at 1,000 μmol photons m^−2^ s^−1^ (SL), specific carotenoids, namely, zeaxanthin, echinenone, and myxoxanthophyll, accumulated at high levels, and the photoinhibition of PSII was less marked than in cells that had been grown under standard growth light at 70 μmol photons m^−2^ s^−1^ (GL). The rate of photodamage to PSII, as monitored in the presence of lincomycin, did not differ between cells grown under SL and GL, suggesting that the mitigation of photoinhibition after acclimation to SL might be attributable to the enhanced ability to repair PSII. When cells grown under GL were transferred to SL, the mitigation of photoinhibition of PSII occurred in two distinct stages: a first stage that lasted 4 h and the second stage that occurred after 8 h. During the second stage, the accumulation of specific carotenoids was detected, together with enhanced synthesis *de novo* of proteins that are required for the repair of PSII, such as the D1 protein, and suppression of the production of singlet oxygen (^1^O_2_). In the Δ*crtR*Δ*crtO* mutant of *Synechocystis*, which lacks zeaxanthin, echinenone, and myxoxanthophyll, the mitigation of photoinhibition of PSII, the enhancement of protein synthesis, and the suppression of production of ^1^O_2_ were significantly impaired during the second stage of acclimation. Thus, elevated levels of the specific carotenoids during acclimation to strong light appeared to protect protein synthesis from ^1^O_2_, with the resultant mitigation of photoinhibition of PSII.

## Introduction

Light is necessary for photosynthesis but excess light impairs photosynthesis. Photosystem II (PSII), which is a protein-pigment complex that converts light energy to chemical energy, is known to be particularly sensitive to strong light. Exposure of photosynthetic organisms to strong light results in the specific inactivation of PSII, and this phenomenon is referred to as photoinhibition of PSII (Aro et al., 1993b; Vass, 2012). Photoinhibition of PSII becomes apparent under strong light when the rate of photodamage to PSII exceeds the rate of repair of PSII (Murata and Nishiyama, 2018). Photodamaged PSII is repaired *via* an efficient repair system that involves proteolytic degradation of damaged D1 protein; synthesis *de novo* of the precursor to the D1 protein (pre-D1); insertion of the pre-D1 into PSII; processing of pre-D1 at the carboxy-terminal extension; and reactivation of PSII (Theis and Schroda, 2016; Li et al., 2018).

Photodamage to PSII depends on the intensity of incident light (Allakhverdiev and Murata, 2004), whereas the repair of PSII is adversely affected by various types of environmental stress and, in particular, by oxidative stress due to reactive oxygen species (ROS), such as the superoxide anion radical, hydrogen peroxide, the hydroxyl radical, and singlet oxygen (^1^O_2_: Nishiyama et al., 2001; Nishiyama et al., 2004; Allakhverdiev and Murata, 2004). These ROS are produced in abundance in the photosynthetic machinery under strong light, as a result of the transport of electrons and the transfer of excitation energy (Asada, 1999). In the cyanobacterium *Synechocystis* sp. PCC 6803 (hereafter, *Synechocystis*), the ROS-induced inhibition of the repair of PSII has been attributed to the inhibition of synthesis of pre-D1 during translational elongation (Nishiyama et al., 2001; Nishiyama et al., 2004). Biochemical studies revealed that two translation factors, EF-Tu and EF-G, key proteins that support translational elongation, are inactivated *via* oxidation by ROS of specific cysteine residues (Kojima et al., 2007; Yutthanasirikul et al., 2016). Expression, in *Synechocystis*, of mutated EF-Tu or mutated EF-G in which one of the ROS-sensitive cysteine residues had been replaced by a serine residue mitigated the photoinhibition of PSII *via* the acceleration of synthesis *de novo* of proteins, including the D1 protein, with the enhancement of the repair of PSII under strong light (Ejima et al., 2012; Jimbo et al., 2018). Thus, the sensitivity of the repair system to ROS appears to be a critical factor that determines the extent of photoinhibition of PSII.

To minimize levels of ROS, photosynthetic organisms have evolved various anti-oxidative systems, which incorporate ROS-scavenging enzymes and antioxidants. Defects in the anti-oxidative systems exacerbate the photoinhibition of PSII. For example, in mutants of *Synechocystis* that were deficient in catalase and thioredoxin peroxidase, in zeaxanthin and echinenone, and in α-tocopherol, respectively, the photoinhibition of PSII was accelerated as a consequence of the decelerated repair of PSII (Nishiyama et al., 2001; Inoue et al., 2011; Kusama et al., 2015). By contrast, in a mutant of *Synechocystis* that overexpressed superoxide dismutase and catalase, the photoinhibition of PSII was mitigated, with the accelerated repair of PSII (Sae-Tang et al., 2016). In addition, overexpression in *Synechocystis* of orange carotenoid protein, which dissipates excitation energy and depresses the production of ^1^O_2_, protected the repair of PSII under strong light, with the resultant mitigation of photoinhibition of PSII (Takahashi et al., 2019). Thus, the capacity to minimize levels of ROS appears to be essential for the efficient repair of PSII under strong light.

Photosynthetic organisms exhibit enhanced tolerance of PSII to photoinhibition when they acclimate to strong light (Adams et al., 1987). This ability has been associated with the enhanced ability to repair PSII in plants (Aro et al., 1993a), in algae (Erickson et al., 2015), and in cyanobacteria (Samuelsson et al., 1987; Jimbo et al., 2019). During acclimation to strong light, various physiological changes occur (Muramatsu and Hihara, 2012). In cyanobacteria, such changes include a reduction in the size of the antenna phycobilisomes (Grossman et al., 1993), stimulation of the state transition and thermal dissipation of excitation energy (Fujimori et al., 2005), and activation of the Calvin-Benson cycle (Hihara et al., 1998) and anti-oxidative systems (Latifi et al., 2009). With respect to the repair of PSII, it seems likely that activation of anti-oxidative systems might contribute significantly to the enhanced ability to repair PSII during acclimation to strong light. It has been reported that cyanobacteria accumulate specific carotenoids, namely, zeaxanthin, echinenone, and myxoxanthophyll, at high levels when they are grown under strong light, such as 1,300 µmol photons m^−2^ s^−1^ (Masamoto and Furukawa, 1997; Steiger et al., 1999), but the roles of such carotenoids in acclimation to strong light remain to be elucidated.

In the present study, we investigated the roles of carotenoids in the protection of PSII from photoinhibition during acclimation to strong light in the Δ*crtR*Δ*crtO* mutant of *Synechocystis*. In this mutant, zeaxanthin, echinenone, and myxoxanthophyll are deficient as a result of inactivation of genes for β-carotene hydroxylase CrtR, which converts β-carotene and deoxymyxoxanthophyll to zeaxanthin and myxoxanthophyll, respectively, and β-carotene ketolase CrtO, which converts β-carotene to echinenone (Fernández-González et al., 1997; Masamoto et al., 1998; Domonkos et al., 2013). When cells were transferred from growth light to strong light, the photoinhibition of PSII was mitigated in two stages: the first stage occurred within 4 h and the second stage occurred after 8 h. The second stage of mitigation was associated with the accumulation of zeaxanthin, echinenone, and myxoxanthophyll, which contributed to the enhanced repair of PSII *via* suppression of the production of ^1^O_2_ and acceleration of the synthesis *de novo* of proteins that are required for the repair of PSII, such as the D1 protein.

## Materials and Methods

### Cell and Culture Conditions

Cells of a glucose-tolerant strain (hereafter referred to as wild-type) and of the Δ*crtR*Δ*crtO* mutant strain of *Synechocystis* sp. PCC 6803 (Kusama et al., 2015) were grown photoautotrophically at 32°C in liquid BG11 medium under standard growth light at 70 μmol photons m^−2^ s^−1^ (GL), moderately strong light at 200 μmol photons m^−2^ s^−1^ (ML), or strong light at 1,000 μmol photons m^−2^ s^−1^ (SL), with aeration by sterile air that contained 1% (v/v) CO_2_. Cells in cultures with an optical density at 730 nm of 1.0 ± 0.1 were used for assays unless otherwise noted.

### Analysis of Carotenoids

Pigments were extracted from cells with a mixture of acetone and methanol (7:2, v/v) and were analyzed by HPLC on a system equipped with a μBondapak C18 column (8 mm × 100 mm; RCM type; Waters, Milford, MA, U.S.A.), as described previously (Kusama et al., 2015), with slight modifications. Carotenoids were eluted with a linear gradient from a mixture of methanol and water (9:1, v/v) to 100% methanol for 20 min and then with isocratic 100% methanol, at a rate of 1.8 ml min^−1^. Zeaxanthin and β-carotene were detected at 450 nm, while echinenone, myxoxanthophyll, and synechoxanthin were detected at 470 nm. Levels of individual carotenoids were normalized by the content of chlorophyll *a*.

### Assay of Photoinhibition of PSII

For standard assay of photoinhibition, 30-ml aliquots of cell cultures were exposed to light at 2,000 μmol photons m^−2^ s^−1^ at 32°C for designated periods of time to induce the photoinhibition of PSII. For assays of photodamage, lincomycin was added to suspensions of cells at a final concentration of 200 μg ml^−1^ just before the onset of illumination. The activity of PSII was measured at 32°C in the terms of the evolution of oxygen in the presence of 1 mM 1,4-benzoquinone and 1 mM K_3_Fe(CN)_6_ with a Clark*-*type oxygen electrode (Hansatech Instruments, Norfolk, U.K.). For time-course assays after the shift from GL to SL, 30-mL suspensions of cells grown under GL with an optical density at 730 nm of 0.4 ± 0.1 were incubated under SL and aliquots of 1 mL were withdrawn at designated times for measurements of the activity of PSII. Aliquots of 1 mL were illuminated with strong light at 1,500 µmol photons m^−2^ s^−1^ for 30 min at 32°C within the chamber of the oxygen electrode and the activity of PSII was measured. The ratio, as a percentage, of the residual activity of PSII to the initial activity of PSII at each designated time point was defined as the strong-light tolerance of PSII.

### Quantitation of Chlorophyll and Carotenoids

Chlorophyll *a* and carotenoids were extracted from cells with 100% methanol and the concentrations of these pigments were determined spectroscopically, as described previously (Wellburn, 1994; Ritchie, 2006).

### Detection of ^1^O_2_

The production of ^1^O_2_ in cells was detected by measuring the rate of the light-induced uptake of oxygen in the presence of histidine, as described previously (Rehman et al., 2013; Kusama et al., 2015). Cells in cultures with an optical density at 730 nm of 0.5 ± 0.1 were exposed to light at 2,500 μmol photons m^−2^ s^−1^ at 32°C in the presence of 5 mM histidine and in its absence, and the evolution of oxygen was measured in the absence of electron acceptors. The generation of ^1^O_2_ was quantitated by subtracting the rate of the evolution of oxygen in the absence of histidine from the rate in its presence. Assays were also performed in the presence of either 10 µM DCMU or 10 mM NaN_3_.

### Labeling of Proteins *In Vivo*

For pulse labeling of proteins, 15-ml aliquots of cell cultures were incubated at 32°C in light at 1,500 µmol photons m^-2^ s^-1^ for 15 min in the presence of 240 kBq ml^−1^ ^35^S-labeled methionine plus cysteine (EasyTag™ EXPRE^35^S^35^S; PerkinElmer, Waltham, MA, U.S.A.), as described previously (Nishiyama et al., 2004). Labeling was terminated by the addition of non-radioactive methionine and cysteine to a final concentration of 2 mM each, with immediate cooling of samples on ice. Thylakoid membranes were isolated from cells as described previously (Nishiyama et al., 2004), and membrane proteins were separated by SDS-PAGE on a 12.5% polyacrylamide gel that contained 6 M urea. Labeled proteins on the gel were visualized with an imaging analyzer (FLA-7000; Fujifilm, Tokyo, Japan) and levels of the D1 protein were determined densitometrically, as described previously (Kojima et al., 2007).

## Results

### Effects of Specific Carotenoids on Growth Under Strong Light

**Figure 1:**
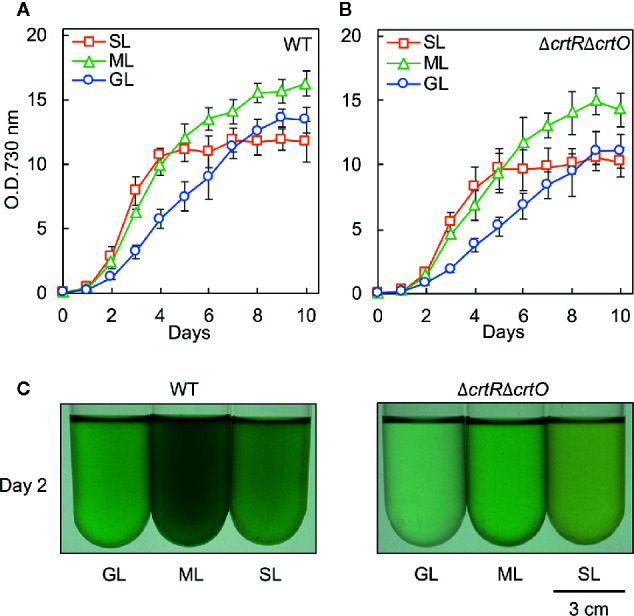
Growth of wild-type and Δ*crtR*Δ*crtO* cells under light at three different intensities. Cells were grown photoautotrophically at 32°C under standard growth light (GL) at 70 μmol photons m^−2^ s^−1^, under moderately strong light (ML) at 200 μmol photons m^−2^ s^−1^, and under strong light (SL) at 1,000 μmol photons m^−2^ s^−1^. Growth curves of wild-type **(A)** and Δ*crtR*Δ*crtO* **(B)** cells as monitored in terms of optical density at 730 nm. **(C)** Representative images of cell suspensions after culture for 2 days. Values are means ± SD (bars) of results from three independent experiments.

We grew cells of the wild-type strain of *Synechocystis* and the derivative Δ*crtR*Δ*crtO* strain, which is deficient in zeaxanthin, echinenone, and myxoxanthophyll (Kusama et al., 2015), under standard growth light (GL; 70 µmol photons m^−2^ s^−1^), moderately strong light (ML; 200 µmol photons m^−2^ s^−1^), and strong light (SL; 1,000 µmol photons m^−2^ s^−1^). Wild-type cells grew faster under ML than under GL (**Figure 1A**). Under SL, wild-type cells grew at the almost same rate as under ML during the first 4 d and then the proliferation of cells ceased (**Figure 1A**). The growth of Δ*crtR*Δ*crtO* cells was slower than that of wild-type cells under light at the three different intensities and exhibited light dependency similar to that of wild-type cells (**Figure 1B**). Under SL, suspensions of both types of cell looked yellower in color than under GL and ML (**Figure 1C**). Spectroscopic analyses showed that under SL, the contents of chlorophyll *a* and phycocyanin decreased in both types of cell, while the contents of carotenoids increased (**Supplementary Figure S1**, **Supplementary Table S1**). The most striking difference between the two strains was that wild-type cells remained blue-green in color for 10 d under SL, whereas Δ*crtR*Δ*crtO* cells started to bleach within 8 to 10 d under SL, indicating that the mutant was sensitive to strong light.

### Levels of Carotenoids in Cells Grown Under Strong Light

**Figure 2:**
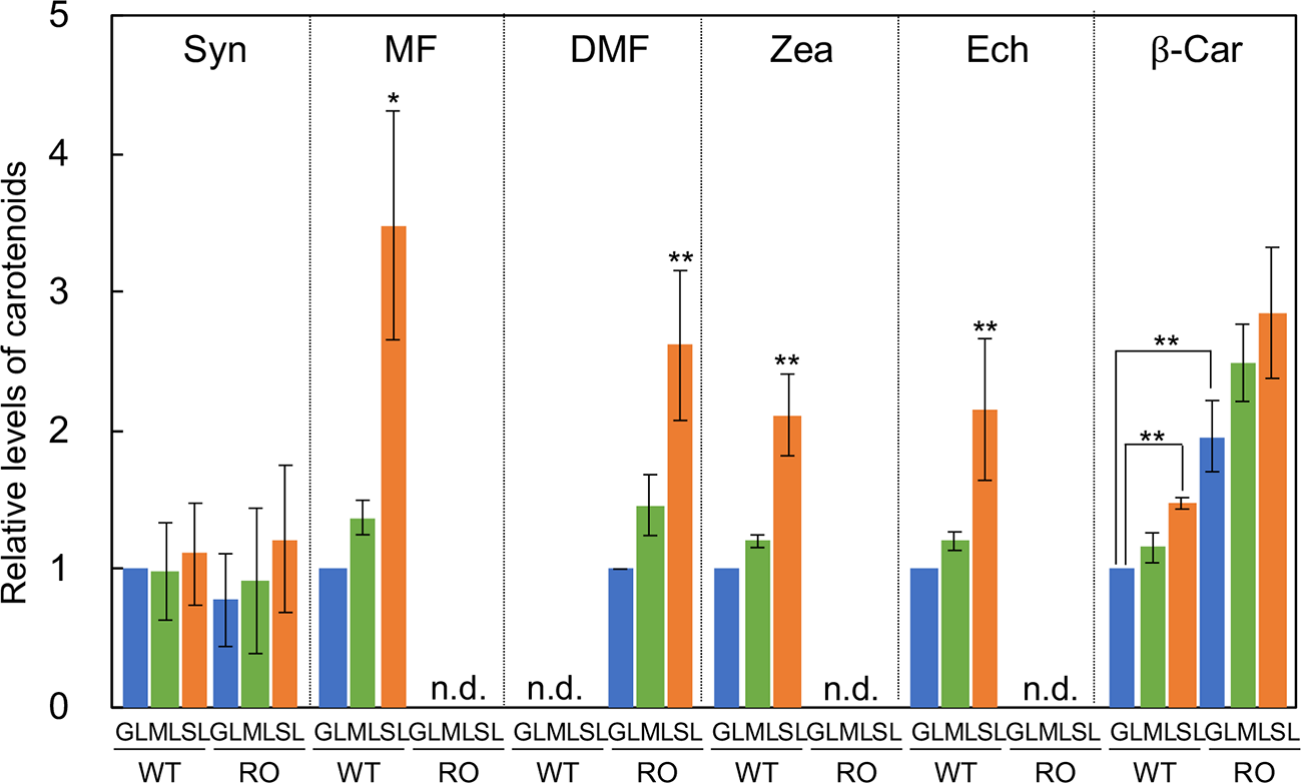
Levels of carotenoids in wild-type (WT) and Δ*crtR*Δ*crtO* (RO) cells grown under GL, ML and SL (see legend to **Figure 1**). Syn, synechoxanthin; MF, myxoxanthophyll; DMF, deoxymyxoxanthophyll; Zea, zeaxanthin; Ech, echinenone; β-Car, β-carotene; n.d., not detected. Levels of Syn, MF, Zea, Ech, and β-Car are relative to those in wild-type cells grown under GL, respectively, while levels of DMF are relative to that in Δ*crtR*Δ*crtO* cells grown under GL. Values are means ± SD (bars) of results from three independent experiments. Asterisks indicate statistically significant differences (*P < 0.05; **P < 0.01; Student's *t* test).

The major carotenoids in *Synechocystis* are β-carotene, zeaxanthin, echinenone, and myxoxanthophyll (Takaichi and Mochimaru, 2007; Kusama et al., 2015). We analyzed the levels of these carotenoids in wild-type and Δ*crtR*Δ*crtO* cells that had been grown under GL, ML, and SL for 24 h (**Figure 2**). In wild-type cells grown under SL, levels of β-carotene, zeaxanthin, echinenone, and myxoxanthophyll were much higher than those in cells grown under GL and ML. There was no detectable zeaxanthin, echinenone or myxoxanthophyll in Δ*crtR*Δ*crtO* cells. However, in Δ*crtR*Δ*crtO* cells grown under SL, levels of deoxymyxoxanthophyll and β-carotene were higher than those in cells grown under GL and ML. Since myxoxanthophyll is synthesized from deoxymyxoxanthophyll *via* a reaction catalyzed by β-carotene hydroxylase CrtR (Takaichi et al., 2001), it is reasonable that deoxymyxoxanthophyll accumulated in Δ*crtR*Δ*crtO* cells. The level of β-carotene in Δ*crtR*Δ*crtO* cells was about twice that in wild-type cells. Synechoxanthin, a carotenoid found specifically in cyanobacteria (Graham and Bryant, 2008), accumulated at very low levels in both types of cell under all light conditions tested.

### Specific Carotenoids Protect the Repair of PSII During Acclimation to Strong Light

**Figure 3:**
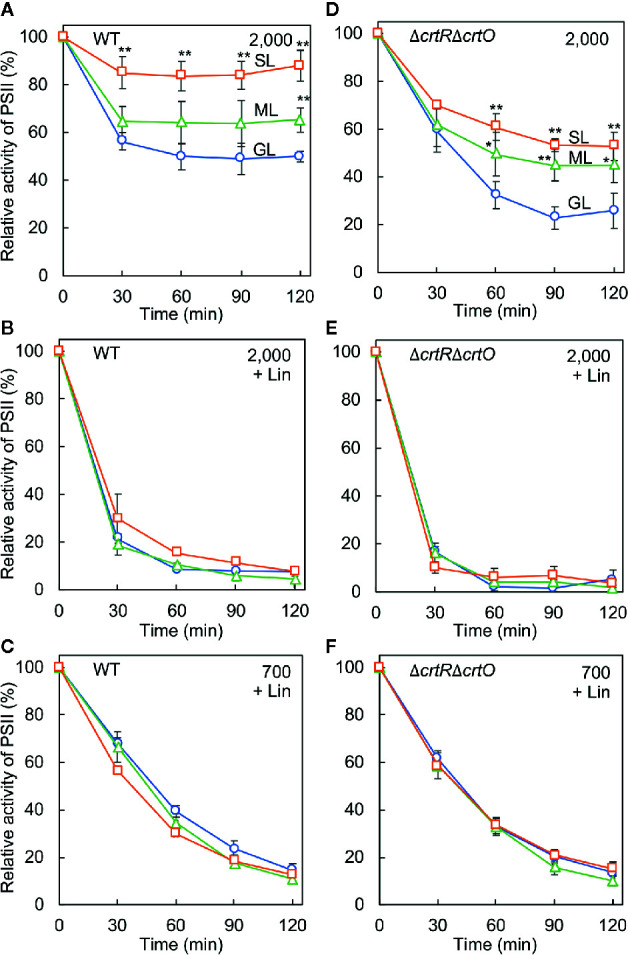
Effects of growth light on the photoinhibition of PSII in wild-type **(A–C)** and Δ*crtR*Δ*crtO* **(D–F)** cells. Cells that had been grown under GL, ML, and SL (see legend to **Figure 1**) for 24 h were exposed to strong light at 2,000 µmol photons m^−2^ s^−1^, with aeration by ambient air, in the absence of lincomycin (Lin; **A, D**) and in its presence **(B, E)**. Cells were also exposed to strong light at 700 µmol photons m^−2^ s^−1^ in the presence of lincomycin **(C, F)**. The activity of PSII was monitored in terms of the evolution of oxygen in the presence 1 mM 1,4-benzoquinone as the electron acceptor. The activities taken as 100% for wild-type cells grown under GL, ML, and SL were 1,075 ± 118, 1,060 ± 144, 1,006 ± 200 µmol O_2_ mg^−1^ Chl h^−1^, respectively. The activities taken as 100% for Δ*crtR*Δ*crtO* cells grown under GL, ML, and SL were 561 ± 167, 501 ± 71, 482 ± 99 µmol O_2_ mg^−1^ Chl h^−1^, respectively. Values are means ± SD (bars) of results from five independent experiments. Asterisks indicate statistically significant differences compared to cells grown under GL (*P < 0.05; **P < 0.01; Student's *t*-test).

We examined the photoinhibition of PSII in wild-type and Δ*crtR*Δ*crtO* cells that had been grown under GL, ML, and SL to an optical density at 730 nm of 0.8 ± 0.1. When wild-type cells grown under GL were exposed to light at 2,000 µmol photons m^−2^ s^−1^, the activity of PSII fell to 51% of the initial level in 120 min (**Figure 3A**). By contrast, the activity of PSII in cells grown under ML remained at 65% of the initial level after 120 min, and the activity of PSII in cells grown under SL remained at 88% of the initial level. However, when cells were exposed to light at 2,000 µmol photons m^−2^ s^−1^ in the presence of lincomycin, which blocks the repair of PSII, the activity of PSII in cells grown under GL, under ML, and under SL fell at similar rates (**Figure 3B**). When cells were exposed to a weaker light at 700 µmol photons m^−2^ s^−1^ in the presence of lincomycin, the activity of PSII in cells grown under GL, under ML and under SL also fell at similar rates (**Figure 3C**), suggesting that increasing the intensity of the growth light did not affect photodamage but enhanced the repair of PSII. When Δ*crtR*Δ*crtO* cells that had been grown under GL were exposed to light at 2,000 µmol photons m^−2^ s^−1^, the activity of PSII fell to 26% of the initial level within 120 min (**Figure 3D**). The activity of PSII in Δ*crtR*Δ*crtO* cells that had been grown under ML remained at 45% of initial level after 120 min, and the activity of PSII in cells in Δ*crtR*Δ*crtO* cells that had been grown under SL remained at 53% of the initial level. There were no differences in the extent of photodamage to PSII under light at 2,000 or 700 µmol photons m^−2^ s^−1^ among cells grown under GL, under ML and under SL (**Figures 3E, F**). Thus, it appeared that Δ*crtR*Δ*crtO* cells were more susceptible than wild-type cells to photoinhibition of PSII, as a consequence of the decreased ability to repair PSII. Nevertheless, the ability to enhance the repair of PSII after growth under ML and SL was retained to some extent in Δ*crtR*Δ*crtO* cells.

### Specific Carotenoids Mitigate the Photoinhibition of PSII During the Second Stage of Acclimation to Strong Light

**Figure 4:**
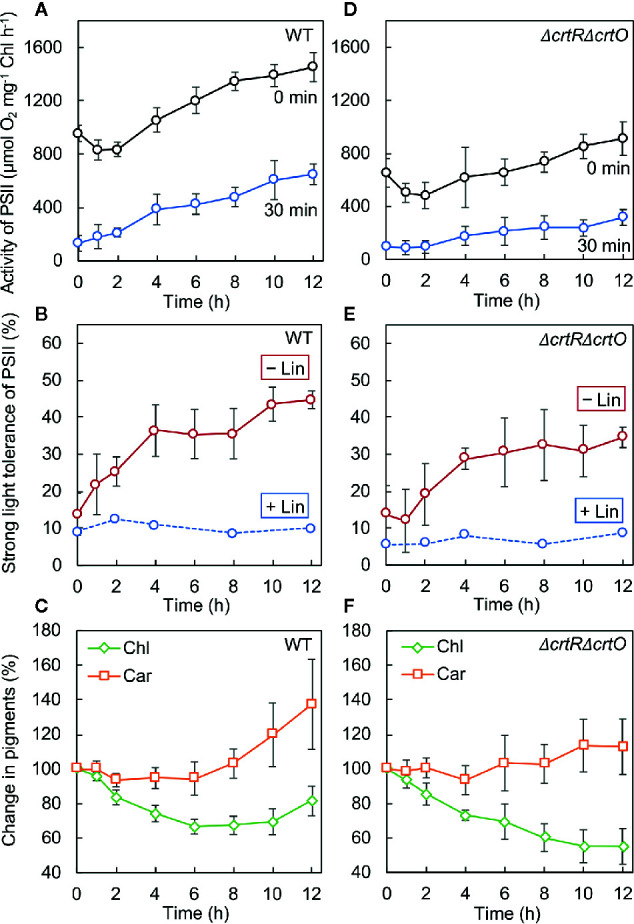
Changes in the activity of PSII, the strong-light tolerance of PSII, and levels of pigments after transfer of wild-type **(A–C)** and Δ*crtR*Δ*crtO* **(D–F)** cells from GL to SL (see legend to **Figure 1**). **(A, D)** Changes in the activity of PSII at the indicated times (0 min) and the residual activity of PSII after cells at the indicated times had been exposed to light at 1,500 µmol photons m^−2^ s^−1^ for 30 min (30 min). **(B, E)** The strong-light tolerance of PSII, defined as the ratio (as a percentage) of the residual activity of PSII (30 min) to the initial activity of PSII (0 min) at the indicated times, as mentioned above. Photodamage to PSII was also monitored by addition of lincomycin (Lin) prior to exposure of cells to light at 1,500 µmol photons m^−2^ s^−1^ for 30 min. **(C, F)** Changes in levels of chlorophyll *a* (Chl) and carotenoids (Car). Values are means ± SD (bars) of results from five independent experiments.

We monitored the time course of changes in the activity of PSII after cells that had been grown under GL were transferred to SL. In wild-type cells, the activity of PSII dropped by 15% in 2 h and then increased to above the initial activity in 4 h (**Figure 4A**, 0 min). We also withdrew aliquots of cell suspensions at designated times during incubation under SL and exposed them to light at 1,500 µmol photons m^−2^ s^−1^ for 30 min to induce the photoinhibition of PSII. The residual activity of PSII increased during incubation under SL (**Figure 4A**, 30 min). The ratio (as a percentage) of the residual activity of PSII to the initial activity of PSII at each designated time point was defined as the strong-light tolerance of PSII. Under SL, the strong-light tolerance of PSII increased from 14% to 36% in 4 h and reached a plateau (**Figure 4B**, −Lin). Then the strong-light tolerance started to increase again after 8 h and reached 45% within 12 h. Thus, it appeared that the mitigation of photoinhibition of PSII occurred in two stages: the first stage occurred during the first 4 h and the second stage occurred after 8 h. However, when the strong-light tolerance of PSII was monitored in the presence of lincomycin, it failed to increase (**Figure 4B**, +Lin), suggesting that the repair of PSII was enhanced during transfer of cells from GL to SL. We also monitored changes in levels of chlorophyll *a* and carotenoids after the transfer of cells to SL. Levels of chlorophyll *a* fell rapidly under SL, whereas levels of carotenoids started to rise within 8 h under SL (**Figure 4C**).

In Δ*crtR*Δ*crtO* cells, the activity of PSII dropped by 21% in 2 h and then increased, albeit more slowly than that in wild-type cells, under SL (**Figure 4D**, 0 min). The residual activity of PSII after exposure of cells to light at 1,500 µmol photons m^−2^ s^−1^ for 30 min was much lower than that in wild-type cells and also increased under SL, but again more slowly than in wild-type cells (**Figure 4D**, 30 min). Under SL, the strong-light tolerance of PSII increased from 14% to 35% and then ceased to increase significantly (**Figure 4E**, −Lin). Moreover, it did not change in the presence of lincomycin (**Figure 4E**, +Lin). These results indicate that the enhancement of the repair of PSII during the second stage might have been impaired in the mutant cells. Levels of chlorophyll *a* continued to decline under SL, while levels of carotenoids remained almost unchanged (**Figure 4F**). Comparison of wild-type cells to Δ*crtR*Δ*crtO* cells suggested that the accumulation of zeaxanthin, echinenone, and myxoxanthophyll might be associated with the enhanced repair of PSII during the second stage.

### Specific Carotenoids Enhance the Synthesis of the D1 Protein During Acclimation to Strong Light

**Figure 5:**
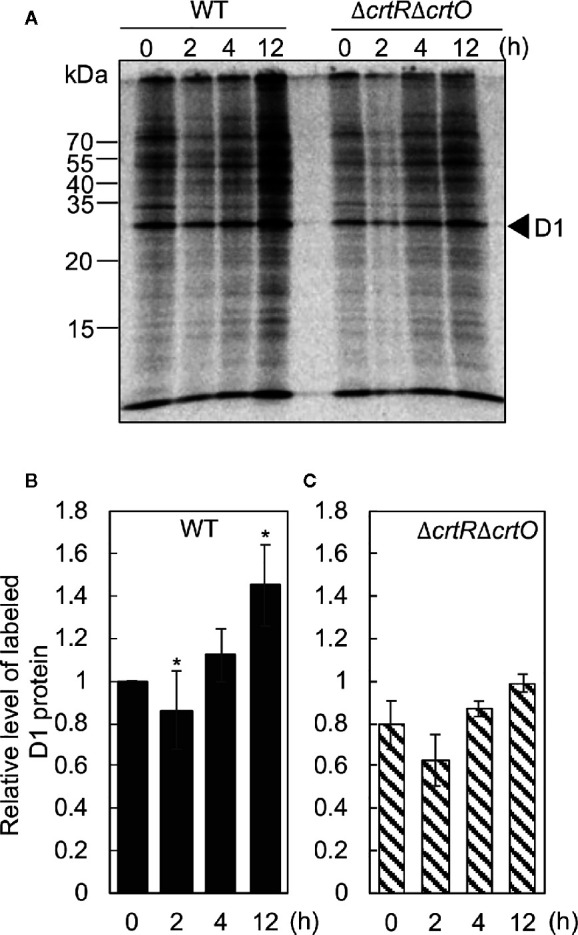
Changes in the synthesis *de novo* of proteins in thylakoid membranes after transfer of wild-type and Δ*crtR*Δ*crtO* cells from GL to SL (see legend to **Figure 1**). Proteins from cells that had been incubated under SL for the indicated times were pulse-labeled by incubation of cells at 32°C, for 15 min, under strong light at 1,500 µmol photons m^−2^ s^−1^ in the presence of ^35^S-labeled methionine plus cysteine. **(A)** A representative radiogram of pulse-labeled proteins from thylakoid membranes. **(B, C)** Quantitation of levels of labeled D1 protein in wild-type **(B)** and Δ*crtR*Δ*crtO* **(C)** cells. Levels of labeled D1 protein were normalized by reference to those in wild-type cells before transfer of cells to SL, namely, at zero time. Values are means ± SD (bars) of results from three independent experiments. Asterisks indicate statistically significant differences compared to the levels at zero time (*P < 0.05; Student's *t*-test).

The synthesis *de novo* of the D1 protein plays a vital role in the repair of PSII (Aro et al., 1993b). To examine the effects of elevated levels of carotenoids on the synthesis *de novo* of the D1 protein, we monitored the incorporation of ^35^S-labeled methionine plus cysteine into proteins during the exposure of cells to strong light at 1,500 µmol photons m^−2^ s^−1^ for 15 min. **Figure 5A** shows the patterns of pulse-labeled proteins from thylakoid membranes of wild-type and Δ*crtR*Δ*crtO* cells after cells had been transferred from GL to SL and incubated for designated times. In wild-type cells, the rate of synthesis of the D1 protein dropped by 11% in 2 h and then increased 1.1- and 1.4-fold by 4 and 12 h, respectively, under SL (**Figure 5B**). In Δ*crtR*Δ*crtO* cells, the rate of synthesis of the D1 protein under SL was 20% lower than in wild-type cells (**Figure 5C**). The rate of synthesis dropped by 20% within 2 h, returned to the initial level at 4 h, and had increased 1.2-fold by 12 h (**Figure 5C**). In particular, the acceleration of synthesis of the D1 protein, as determined after 12 h, was much lower in Δ*crtR*Δ*crtO* cells than in wild-type cells. Note that the patterns of synthesis of almost all the thylakoid proteins were similar to that of the D1 protein, suggesting that the absence of zeaxanthin, echinenone, and myxoxanthophyll had an adverse effect on overall protein synthesis during incubation under SL (**Figure 5A**).

### Specific Carotenoids Depress the Production of ^1^O_2_ During Acclimation to Strong Light

**Figure 6:**
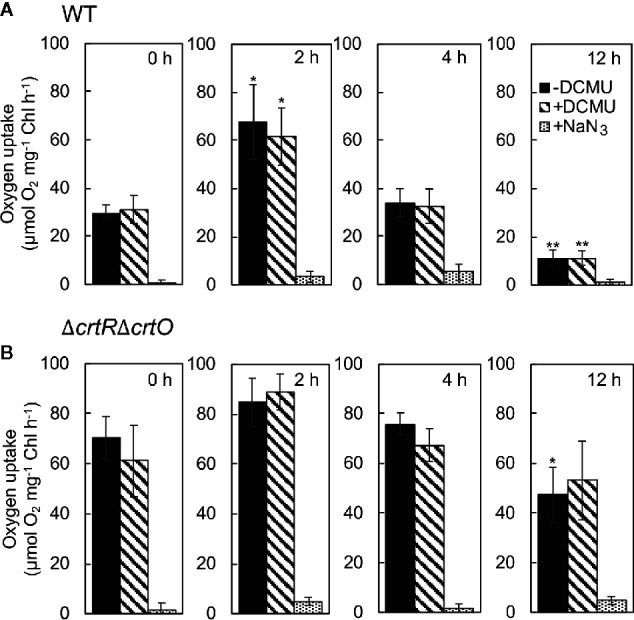
Changes in the rate of production of ^1^O_2_ in wild-type **(A)** and Δ*crtR*Δ*crtO* **(B)** cells after transfer of cells from GL to SL. The production of ^1^O_2_ was monitored in terms of the difference between uptake of O_2_ in the presence of histidine and in its absence under strong light at 2,500 µmol photons m^−2^ s^−1^. The uptake of O_2_ was measured in the absence of DCMU, in the presence of 10 µM DCMU, and in the presence of 10 µM DCMU plus 10 mM NaN_3_. Values are means ± SD of results from three independent experiments. Asterisks indicate statistically significant differences compared to the levels at zero time (*P < 0.05; **P < 0.01; Student's *t* test).

Zeaxanthin, echinenone, and myxoxanthophyll are effective scavengers of ^1^O_2_ (Young and Frank, 1996; Sandmann, 2019), and the scavenging abilities of these carotenoids are associated with the repair of PSII (Kusama et al., 2015). To monitor changes in levels of ^1^O_2_ during acclimation to strong light, we measured the rates of production of ^1^O_2_ by cells under strong illumination at 2,500 µmol photons m^−2^ s^−1^ in terms of the light-induced uptake of O_2_ in the presence of histidine (Kusama et al., 2015). When wild-type cells were transferred to SL, the rate of production of ^1^O_2_ increased 2.3-fold within 2 h; it returned to the initial level within 4 h; and it had fallen to 37% of the initial level by 12 h (**Figure 6A**). The production of ^1^O_2_ was unaffected by 3-(3,4-dichlorophenyl)-1,1-dimethylurea (DCMU), which blocks the photosynthetic transport of electrons, while it was abolished in the presence of NaN_3_, a quencher of ^1^O_2,_ confirming the accurate detection of ^1^O_2_, as reported previously (Kusama et al., 2015). In Δ*crtR*Δ*crtO* cells, the rate of production of ^1^O_2_ was 2.4 times higher than that in wild-type cells before the transfer of cells to SL (**Figure 6B**). After the transfer of cells from GL to SL, the rate of production of ^1^O_2_ increased 1.2-fold within 2 h, returned to the initial level within 4 h, and decreased to 67% of the initial level within 12 h (**Figure 6B**). Thus, it appeared that the presence of zeaxanthin, echinenone, and myxoxanthophyll might depress the production of ^1^O_2_ under strong light and, also, that the accumulation of these carotenoids during acclimation to strong light might suppress the production of ^1^O_2_ to an even greater extent.

## Discussion

### Involvement of Specific Carotenoids in the Protection of the Repair of PSII During Acclimation to Strong Light

Earlier studies found that mitigation of the photoinhibition of PSII during acclimation to strong light is associated with the enhanced repair of PSII in plants (Aro et al., 1993a), algae (Erickson et al., 2015), and cyanobacteria (Samuelsson et al., 1987; Jimbo et al., 2019). It was also reported that carotenoids are required for the assembly and photoprotection of PSII (Sozer et al., 2010; Hakkila et al., 2013). The present study revealed that the accumulation of specific carotenoids, namely, zeaxanthin, echinenone, and myxoxanthophyll, during acclimation to strong light is involved in the enhanced repair of PSII, with the resultant mitigation of photoinhibition of PSII in *Synechocystis*. However, even in the absence of such carotenoids, Δ*crtR*Δ*crtO* cells were able to enhance the repair of PSII to some extent when grown under strong light (**Figure 3**). These results indicate that not only the accumulation of the specific carotenoids but also some other mechanism(s) might be responsible for the optimally enhanced repair of PSII during acclimation to strong light. Nevertheless, the significant decrease in the capacity for repair of PSII in Δ*crtR*Δ*crtO* cells suggests that these specific carotenoids might play a crucial role in protection of the repair of PSII from inhibition under strong light, thereby allowing cells to survive under such conditions (**Figure 1**).

### Mechanism of the Mitigation of Photoinhibition of PSII During Acclimation to Strong Light

The mitigation of photoinhibition of PSII, namely, the enhancement of repair of PSII, occurred in two distinct stages: the first stage occurred during the first 4 h, and the second stage occurred after 8 h (**Figure 4**). During the second stage, specific carotenoids, namely, zeaxanthin, echinenone, and myxoxanthophyll, accumulated at high levels, while the synthesis *de novo* of proteins, including the D1 protein, was activated, and the production of ^1^O_2_ was suppressed. Conversely, in the absence of these carotenoids, the second stage of mitigation of photoinhibition of PSII, the activation of protein synthesis, and the suppression of production of ^1^O_2_ were significantly impaired. These observations together suggest a mechanism by which carotenoids might protect the repair of PSII during acclimation to strong light as follows. High levels of accumulation of the specific carotenoids during the second stage of acclimation might protect protein synthesis by depressing the production of ^1^O_2_, with the resultant enhanced repair of PSII.

By contrast, we detected no obvious increase in levels of carotenoids during the first stage of mitigation of photoinhibition of PSII (**Figure 4**). What mechanism might be involved in this first stage of mitigation? Within the first 2 h after cells had been transferred from growth light to strong light, both the activity of PSII and the rate of synthesis of the D1 protein decreased, while the production of ^1^O_2_ rose. These features are typical of photoinhibition of PSII. During the next 2 h under strong light, by contrast, the activity of PSII and the rate of synthesis of the D1 protein rose, while the production of ^1^O_2_ decreased. These changes might involve other mechanisms that protect protein synthesis from photo-oxidative stress. Recent studies of the acclimation of *Synechocystis* to strong light revealed that levels of the translation factor EF-Tu, which is sensitive to oxidation by ROS, rise during acclimation to strong light and that the elevated levels of EF-Tu help to accelerate protein synthesis and enhance the repair of PSII under strong light (Jimbo et al., 2019). It seems likely that, during the first 4 h after transfer of cells from growth light to strong light, levels of EF-Tu might increase and contribute to enhanced repair of PSII. In addition, transfer of cells to strong light should stimulate the production of ATP and reducing power, which in turn enhances the repair of PSII *via* the acceleration of the synthesis of the D1 protein at both transcriptional and translational levels (Murata and Nishiyama, 2018). Thermal dissipation of excitation energy, which is a major component of non-photochemical quenching (NPQ), might also contribute to the enhanced repair of PSII during the first 4 h of acclimation. In *Synechocystis*, exposure of cells to strong light converts orange carotenoid protein (OCP) from its inactive form to its active form, stimulating the thermal dissipation of excitation energy (Wilson et al., 2006) and enhancing the repair of PSII (Takahashi et al., 2019). The impaired mitigation of photoinhibition of PSII in *crtR*Δ*crtO* cells might also be due, in part, to the lack of 3′-hydroxyechinenone, the cofactor of OCP, and the consequent loss of active OCP. Reduction in the size of the antenna complex, the phycobilisomes, might also minimize oxidative stress and enhance the repair of PSII *via* reduction of the transfer of excitation energy to the reaction center (Grossman et al., 1993; Kopečná et al., 2012). All these mechanisms might work together to enhance the repair of PSII not only during the first stage but also during the second stage of acclimation.

### The Physiological Roles of Specific Carotenoids During Acclimation to Strong Light

Zeaxanthin, echinenone, and myxoxanthophyll are effective scavengers of ^1^O_2_ and free radicals (Sandmann, 2019). Their abilities to scavenge ^1^O_2_ in organic solvents are higher than that of β-carotene because of the presence of hydroxyl and glycosyl groups (Sandmann, 2019). These features might explain why these three specific carotenoids accumulate in abundance during acclimation to strong light. In Δ*crtR*Δ*crtO* cells, the level of β-carotene was about twice that in wild-type cells and increased 1.5-fold after acclimation to strong light (**Figure 2**). Nonetheless, the impaired ability to repair PSII in Δ*crtR*Δ*crtO* cells suggests that β-carotene cannot substitute for these three specific carotenoids in terms of the protection of the repair of PSII under strong light.

Most carotenoids are located in thylakoid and cytoplasmic membranes, although their precise localization within these membranes remains to be elucidated (Masamoto et al., 1999; Zhang et al., 2015). It seems likely that carotenoids that are localized within and in close vicinity to the reaction center of PSII quench the triplet state of chlorophyll to prevent the formation of ^1^O_2_, while other carotenoids scavenge ^1^O_2_ directly. Zeaxanthin, echinenone, and myxoxanthophyll at elevated levels are likely to act in this way to depress intracellular levels of ^1^O_2_, which is produced in abundance from PSII during the transfer of excitation energy under strong light.

As mentioned above, Δ*crtR*Δ*crtO* cells did retain some ability to enhance the repair of PSII. We found that, in this mutant, deoxymyxoxanthophyll, a precursor to myxoxanthophyll, accumulated, with its level increasing 2.5-fold after the transfer of cells from growth light to strong light (**Figure 2**). The accumulation of deoxymyxoxanthophyll might, in part, contribute to the enhanced repair of PSII in the absence of zeaxanthin, echinenone, and myxoxanthophyll since this carotenoid also has a glycosyl group. The roles of myxoxanthophyll and deoxymyxoxanthophyll in the protection of the repair of PSII and their localization require further clarification.

## Conclusion

During the acclimation of *Synechocystis* to strong light, specific carotenoids, namely, zeaxanthin, echinenone, and myxoxanthophyll, accumulate in abundance and enhance the repair of PSII, with the resultant mitigation of photoinhibition of PSII. The accumulation of these carotenoids, which occurs at the late stage of acclimation, depresses the production of ^1^O_2_ and thereby protects the synthesis *de novo* of proteins that are required for the repair of PSII, such as the D1 protein, under strong light.

## Data Availability Statement

The datasets generated for this study are available on request to the corresponding author.

## Author Contributions

TI performed most of the experiments. IK examined photoinhibition. HJ supervised the experiments. ST analyzed carotenoids. TI and YN conceived the project and wrote the article, with contributions from all the other authors.

## Funding

This work was supported, in part, by a grant from the MIRAI Program of the Japan Science and Technology Agency (to YN); by a grant from the Japan Society for the Promotion of Science, KAKENHI (grant number JP18K06276 to YN); and by a grant from the Research Program of the “Dynamic Alliance for Open Innovation, Bridging Humans, the Environment and Materials” at the Network Joint Research Center for Materials and Devices (to YN).

## Conflict of Interest

The authors declare that the research was conducted in the absence of any commercial or financial relationships that could be construed as a potential conflict of interest.
