# Supplementary material for: Elevated Levels of Specific Carotenoids During Acclimation to Strong Light Protect the Repair of Photosystem II in Synechocystis sp. PCC 6803
Source: Front Plant Sci. 2020 Jul 7;11:1030. doi: 10.3389/fpls.2020.01030 (PMC7358549; doi:10.3389/fpls.2020.01030)
Supplement: Supplementary file 1 [file DataSheet_1.pdf]

Supplementary Data

Elevated levels of specific carotenoids during acclimation to strong light protect the repair of photosystem II in *Synechocystis* sp. PCC 6803

Taichi Izuhara, Ikumi Kaihatsu, Haruhiko Jimbo, Shinichi Takaichi and Yoshitaka Nishiyama

Supplementary Table S1

Concentration of chlorophyll *a* (Chl *a*) in cells. Wild-type (WT) and Δ*crtR*Δ*crtO* cells were grown for 24 h under growth light at 70 μmol photons m^{-2} s^{-1} (GL), moderately strong light at 200 μmol photons m^{-2} s^{-1} (ML), or strong light at 1,000 μmol photons m^{-2} s^{-1} (SL), and Chl *a* was extracted from cells with 100% methanol. Concentrations of Chl *a* were normalized by optical density at 730 nm ($O.D._{730}$) of cell suspensions. Values are means ± SD (bars) of results from three independent experiments. An asterisk indicates statistically significant difference to cells grown under GL ($P < 0.05$; Student's *t*-test).

	Concentration of Chl *a* (μg Chl mL^{-1} $O.D._{730}^{-1}$)		
	GL	ML	SL
WT	3.3 ± 0.2	3.1 ± 0.7	2.7 ± 0.3*
Δ*crtR*Δ*crtO*	3.1 ± 0.2	2.8 ± 0.5	2.5 ± 0.4

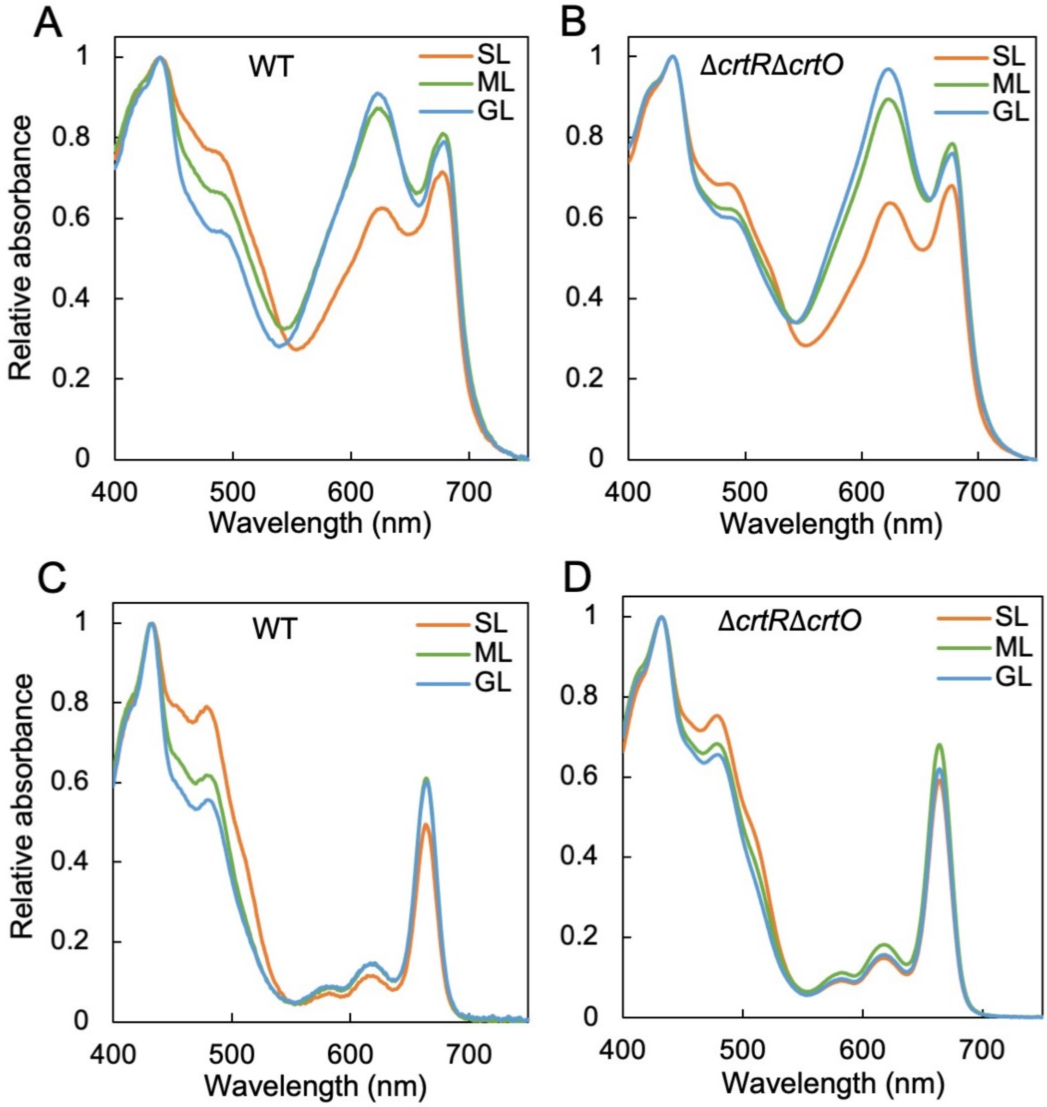

Supplementary Figure S1

Absorption spectra of cell suspensions and extracted pigments. Wild-type (WT) and Δ*crtR*Δ*crtO* cells were grown under GL, ML or SL (see legend to Supplementary Table S1). (A, B) Representative absorption spectra of cell suspensions. (C, D) Representative absorption spectra of pigments that had been extracted from cells with 100% methanol. Spectra were normalized at the peaks at 438 nm and 433 nm for cell suspensions and extracted pigments, respectively. Blue lines, Cells grown under GL; green lines, cells grown under ML; orange lines, cells grown under SL.
